# Protective Effects of *Dendrobium nobile* against Cisplatin Nephrotoxicity Both *In-vitro* and *In-vivo*

**Published:** 2017

**Authors:** Hyeun-Kyoo Shin, Tae-Won Kim, Young-Jung Kim, So-Ra Park, Chang-Seob Seo, Hyekyung Ha, Ju-Young Jung

**Affiliations:** a *Basic Herbal Medicine Research Group, Korea Institute of Oriental Medicine, Yusung- gu, Daejeon, South Korea.*; b *College of Veterinary Medicine & Institute of Veterinary Science, Chungnam National University, Yusung-gu, Daejeon, South Korea.*

**Keywords:** *Dendrobium nobile Lindl*, Acute kidney injury, Cisplatin, p53, Apoptosis

## Abstract

*Dendrobium* genus was reported to contain alkaloid, bibenzyl, fluorenone, phenanthrene, sesquiterpenoid, and phenolic acid, which have biological properties. Our aim was to investigate the protective effect of an aqueous extract of *Dendrobium nobile* Lindl (DNE) against cisplatin-induced acute kidney injury (AKI). Quantification of four phenolic acids (4-hydroxybenzoic, vanillic, syringic, and ferulic acid) in DNE was determined using the HPLC-photodiode array method. Possible protective effects against cisplatin-induced nephrotoxicity were investigated using *in-vitro* (porcine kidney cells; PK15) and *in-vivo* (Sprague Dawley rat) studies. Among the four phenolic acids, 4-hydroxybenzoic acid was the most abundant. In the *in-vitro* study, DNE pretreatment partially prevented decrement of viability after cisplatin (15 μg/mL) treatment in the both the MTT and crystal violet assays. Moreover, relative to cells treated with cisplatin alone, the DNE (50 μg/mL)-pretreated cells showed a ~30% increase in glutathione levels and a ~15% decrease in reactive oxygen species. The expression of p53 was also decreased in DNE-pretreated cells (*p* < 0.05). In the *in-vivo* study, the renal function index decreased to normal levels in groups pretreated with DNE (300 and 500 mg/kg); histopathological alterations and apoptotic cells were also attenuated. Moreover, DNE pretreatment ameliorated oxidative stress in the kidney, as evidenced by recovered antioxidant enzyme levels and decreased lipid peroxidation. DNE, by decreasing oxidative stress, was found to have a protective effect against cisplatin-induced nephrotoxicity. Based on these findings, DNE might be beneficial when treating cisplatin-induced AKI.

## Introduction

The incidence of acute kidney injury (AKI) is still rising, and the associated mortality has been largely unchanged over the past two decades (1). Due to its rapid progression and life-threatening characteristics, prevention is the most important strategy in AKI treatment. 

Cisplatin (*cis-*Diammineplatium II dichloride), a platinum-containing anticancer drug, is widely used for the solid cancers such as ovarian, and head and neck carcinomas (2). Despite its clinical efficacy, dose related nephrotoxicity limits long-term cisplatin-based chemotherapy, which occurs in about one-third of patient undergoing cisplatin treatment (3). In cisplatin nephrotoxicity, cisplatin could selectively accumulate in the kidney through the mediated transport system and activates multiple signaling pathways, resulting in renal tubule cell necrosis and apoptosis (4, 5). Previous studies have reported that the oxidative stress has been recognized as an important factor that contributes to cisplatin nephrotoxicity and glutathione synthesis inhibition and antioxidant depletion have been regarded as a major pathogenic factors (5, 6). In this aspect, many studies have focused on oxidative stress as a therapeutic target (7, 8).

There has been a growing interest in traditional herbal medicine as a source for novel therapeutic agents for AKI. Numerous compounds from different fruits and plants were tested, such as quercetin, lycopene, and platycodin D, which possess antioxidant capacity without toxic side effects (9-11). *Dendrobium* *nobile* Lindl is an herbal medicine that has been used clinically in China and several Southeastern Asian countries to maintain tonicity of the stomach and promote body fluid production (12). Recently, the *Dendrobium* genus has been found to contain compound, that have biological effects, including alkaloid, bibenzyl, fluorenone, phenanthrene, sesquiterpenoid and phenolic acid (13, 14). Moreover, the polysaccharide fraction of D. *nobile* has been found to have antitumor and antioxidant activities (15, 16). 

The aims of the present study were to: 1) determine the phenolic acid compounds in an aqueous extract of *D.* *nobile* Lindl (DNE) using the HPLC-photodiode array (PDA) method, and 2) explore the protective effect of the DNE against cisplatin-induced AKI using both *in-vitro* and *in-vivo* models.

## Experimental

*Chemicals*

4-Hydroxybenzoic acid (purity, ≥99.0%), syringic acid (≥95.0%), vanillic acid (≥97.0%), cisplatin (≥99.0%), captopril (≥98.0%), and crystal violet solution were purchased from Sigma-Aldrich (St. Louis, MO, USA). Ferulic acid (purity ≥98.0%) was obtained from Wako Pure Chemical Industries, Ltd. (Osaka, Japan). *D. nobile* was purchased from Omniherb (Yeongcheon, Korea). The origin of the sample was confirmed taxonomically by Professor Je-Hyun Lee, Dongguk University, Gyeongju, Republic of Korea. A voucher specimen (NO. KIOM-AO15) was deposited in storage at the Basic Herbal Medicine Research Group, Korea Institute of Oriental Medicine. 

*Preparations of DNE and standard solution*

Dried *D. nobile* (60 g) was extracted with distilled water (600 mL) by reflux for 2 h. The extracted solution was filtered through filter paper, evaporated to dryness and freeze-dried (5.19 g). The yield of the water extract obtained was 17.5%. A lyophilized sample (20 mg) was dissolved in distilled water (10 mL) and mixed. The solution was filtered through a SmartPor GHP syringe filter (0.2 μm pore size, Woongki Science, Seoul, Korea). The stock solutions of the four reference standards were dissolved in methanol (1.0 mg/mL) and stored below 4°C.

*HPLC analysis*

A Shimadzu LC-20A HPLC system (Shimadzu Co., Kyoto, Japan) with a PDA detector was employed in this study. The data were processed using LCSolution software (Version 1.24; Shimadzu Co., Kyoto, Japan). The analytical column used for separation was a Gemini C18 column (250 × 4.6 mm; particle size 5 μm; Phenomenex, Torrance, CA, USA) and was maintained at 40°C. The mobile phases for chromatographic separation were carried out using a gradient elution of solvent A (1.0% v/v aqueous acetic acid) and solvent B (1.0% v/v acetic acid in acetonitrile). The gradient flow of the two-solvent system was as follows: 5% B (0 min), 5-70% B (40 min), 70-100% B (45 min), 100% B (50 min), 100-5% B (55 min), and 5% B (70 min). Analysis was performed at a flow-rate of 1.0 mL/min with a detection wavelength of 254 nm. The injection volume was 10 μL.

*Cell viability assay*

For all assays, PK15 cells were seeded at a density of 1 × 10^4^ cells/mL in 96-well plates with regular growth medium. The experiments were carried out on the following day. The effect of DNE on the cisplatin-treated PK15 cells was assessed using MTT and crystal violet assays. PK15 cells were treated with ascorbic acid (positive control, 1.7 mg/mL) and DNE (0, 50, 100 and 200 µg/mL) 2 h before cisplatin (15 µg/mL) treatment; after cisplatin treatment, the cells were incubated for 24 h. The MTT assay was performed using the EZ-Cytox Cell Viability assay kit (Daeil, Seoul, Korea) according to the manufacturer’s protocol. The crystal violet assay was carried out based on a previous report with minor modifications (17). 

*Reactive oxygen species and glutathione levels in PK15 cells*

For both the reactive oxygen species (ROS) and the glutathione (GSH) assays, cells were pretreated with ascorbic acid (positive control, 1.7 mg/mL) and DNE (0 and 50 µg/mL). After 2 h, the cells were treated with cisplatin (15 µg/mL) and incubated for 24 h. The ROS contents were determined using the dihydrodichlorofluoroscein diacetate (Invitrogen, Carlsbad, CA, USA) method with some modification (18). The level of GSH was determined with a Glutathione Assay Kit (Northwest, WA, USA) according to the manufacturer’s protocol.

*Western blot of p53*

PK15 cells were prepared in the same manner as described for the ROS and GSH assays. After incubation, the cells were lysed with lysis buffer (20 mM Tris-HCl pH 8, 150 mM NaCl, protease inhibitor cocktail). After the protein assay, a 30 µg protein sample was separated using 12% SDS-PAGE. The proteins were transferred onto a nitrocellulose membrane in a Semi-Dry Transfer system from Bio-Rad (Hercules, CA, USA). After blocking, the membrane was incubated with primary antibody anti-p53 (1:300; Santa Cruz Biotechnologies, Santa Cruz, CA, USA). Horseradish peroxidase-conjugated anti-mouse IgG (1:5000; Santa Cruz Biotechnologies) was used for p53 detection. Immunoreactivity was visualized using an enhanced chemiluminescence detection kit (Amersham Pharmacia Biotech, Piscataway, NJ, USA).

*Animal study*

Four-week-old male Sprague Dawley (SD) rats were obtained from Orient Bio (Seongnam, Korea) and acclimated to laboratory conditions (25 ± 0.2°C, 50% relative humidity, 12 h light/dark cycle) for 1 week before experimentation. All animals were supplied with standard chow (Charles River Inc., Richmond, IN, USA) and water *ad libitum*. Healthy SD rats (n = 30) were randomly allocated into 6 groups (n = 5/group) and treated for 28 days with each compound orally as follows: group 1 (control, distilled water; DW), group 2 (cisplatin alone, DW + cisplatin), group 3 (100 mg/kg of captopril + cisplatin), groups 4-6 (100, 300 and 500 mg/kg of DNE + cisplatin). Captopril, angiotensin-converting enzyme inhibitor, was used as a positive control drug, which is known to alleviate cisplatin-induced nephrotoxicity by its antioxidant properties and renin-angiotensin system inhibitory effect (19, 20). On day 23, cisplatin (5 mg/kg) was injected intraperitoneally to induce AKI, except for the control group. On day 27, the urine of each animal was collected over 24 h for urine volume analysis; 3 h after the last treatment, the animals were anesthetized with an intraperitoneal injection of a combination of zolazepam and tiletamine (Zoletil; Virbac). The experimental protocols were approved (No. CNU-00070) by the Institutional Animal Care and Use Committee of Chungnam National University (Daejeon, Korea). Blood samples were collected from the inferior vena cava and separated by centrifugation at 800 *g* for 15 min, and the serum blood urea nitrogen (BUN) and creatinine (CRE) were determined on a dry chemistry system (IDEXX Laboratories, Westbrook, ME, USA). The left kidney was quickly removed for histopathological analysis. The other kidney was removed and used in the GSH (Northwest, WA, USA) and the malondialdehyde (MDA; Northwest, WA, USA) assays. The assays were conducted according to the manufacturer’s protocol.

*Histopathological examination*

The left kidney was fixed immediately in a 10% buffered formalin phosphate solution, embedded in paraffin and cut into 5 μm sections and processed for histological staining. These serial tissue sections were either stained with haematoxylin and eosin (H&E) for histopathological examination or subjected to TUNEL staining. Apoptotic nuclei were detected by the TUNEL method using an apoptosis detection kit (Millipore, Bilerica, MA, USA) according to the manufacturer’s protocol. All of the stained slides were analyzed under the light microscope.

*Statistical analysis*

Data are presented as mean values ± standard error of mean (SEM). Significant differences among the experimental groups were determined using the one-way analysis of variance (ANOVA) test followed by Tukey’s post hoc analysis. Values of *P* < 0.05 were considered to be statistically significant.

## Results

*HPLC analysis*

**Figure 1:**
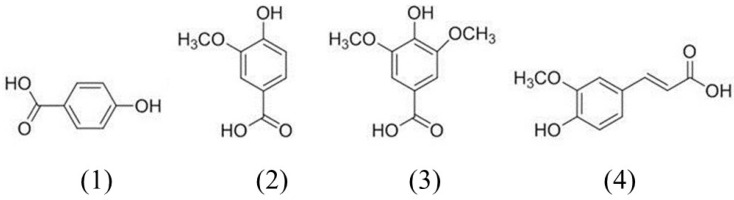
Chemical structures of the phenolic acid compounds in *Dendrobium nobile* Lindl aqueous extracts. 1) 4-hydroxybenzoic acid, 2) vanillic acid, 3) syringic acid, 4) ferulic acid

**Figure 2:**
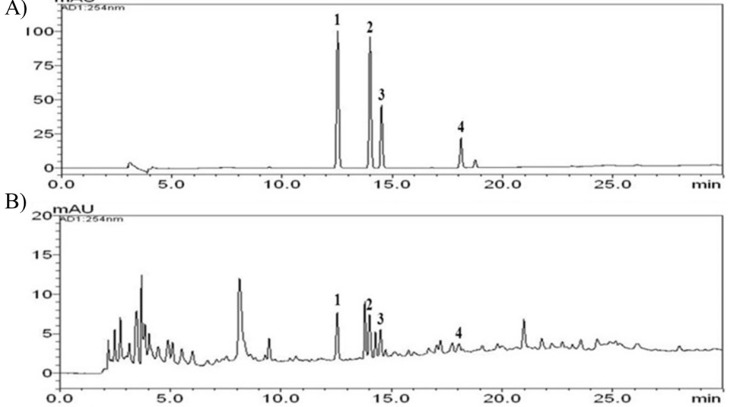
HPLC chromatographs of the standard sample (A) and the standardized *Dendrobium nobile* Lindl aqueous extract (B) measured at a wavelength of 254 nm. The numbers indicate each phenolic acid: 1) 4-hydroxybenzoic acid, 2) vanillic acid, 3) syringic acid, 4) ferulic acid

Figure 1 shows the chemical structures of the four phenolic acids in DNE. Quantitation of the phenolic acids was achieved using PDA detection at 254 nm (Figure 2). The line equations and correlation coefficients (*r*^2^) of the calibration curves and the identified contents of the phenolic acids in DNE are summarized in Table 1. 

**Table 1:** HPLC analysis data for four standard components and the contents of four compounds in the *Dendrobium nobile* Lindl aqueous extract.

Compound	Linear range ( g/mL)	Correlation coefficient (*r*^2^)	LOD ( g/mL)	LOQ ( g/mL)	Contents		
					Mean (mg/g)	SD	RSD (%)
4-hydroxybenzoic acid	0.08-10.00	0.9998	0.014	0.045	3.0	0.06	1.927
Vanillic acid	0.08-10.00	0.9999	0.021	0.068	0.9	0.01	1.567
Syringic acid	0.08-10.00	0.9998	0.012	0.041	1.3	0.02	1.653
Ferulic acid	0.08-10.00	1.0000	0.012	0.041	2.1	0.04	1.857

The limits of detection (LOD) and of quantification (LOQ) were determined using signal-to-noise (S/N) ratios of 3 and 10, respectively.

*In-vitro study*

**Figure 3:**
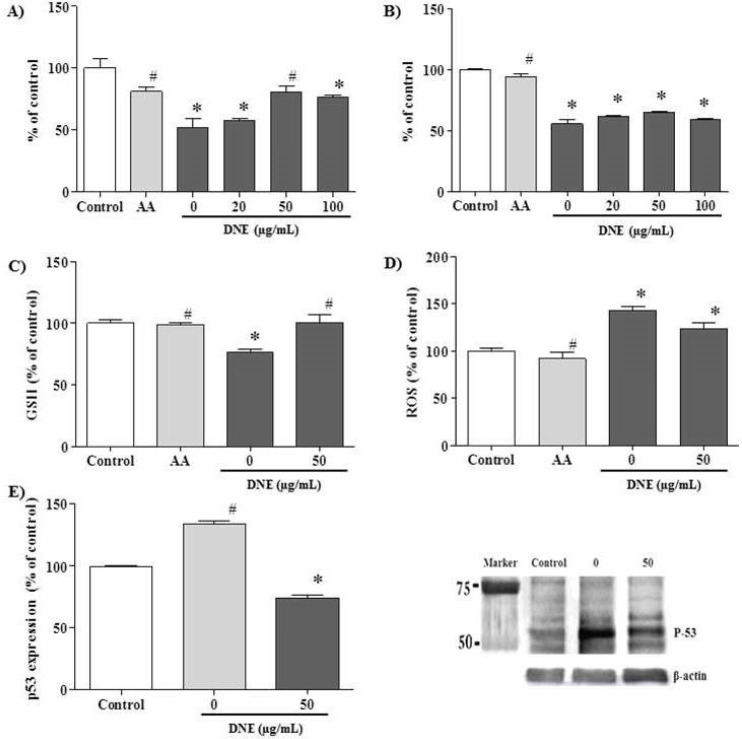
Effect of Dendrobium nobile Lindl extract (DNE) on cisplatin-treated PK15 cells. The cells were treated with ascorbic acid (AA, 1.7 mg/mL) and different concentrations of DNE 2 h before cisplatin (15 μg/mL) treatment, and incubated for 24 h before one of the following assays was performed: A) MTT assay, B) crystal violet assay, C) GSH assay, D) ROS assay, and E) p53 western blot analysis. Values are expressed as mean ± SEM for triplicate experiments. *p < 0.05, a significant difference in comparison with the control cells, #p < 0.05, a significant difference in comparison with cells treated with cisplatin alone

The effect of DNE on cell viability, oxidative status and p53 expression were evaluated in cisplatin-treated PK15 cells (Figure 3). The data are expressed as the percentage relative to the control level. In the viability assay, ascorbic acid (a potent antioxidant used as the positive control) prevented a viability decrement after cisplatin-treatment (*p* < 0.05). DNE pretreatment partially recovered viability when compared to cells treated with cisplatin alone; DNE at 50 μg/mL demonstrated the highest viability in the both MTT (80.8±4.9, *p* < 0.05; Figure 3A) and crystal violet (64.8±0.7, *p* > 0.05; Figure 3B) assays. 

The GSH content was decreased after cisplatin treatment to about 80%; the decreased GSH level was restored almost to the control level in the DNE-pretreated cells (*p* < 0.05; Figure 3C). In the ROS assay, cisplatin treatment increased ROS generation up to about 140% compared to the control group. Treatment with 50 µg/mL DNE slightly decreased cisplatin-induced ROS production to about 130% (*p* < 0.05; Figure 3D). 

In the western blot assay, p53 expression was markedly increased after cisplatin treatment (Figure 3E). However, pretreatment with DNE suppressed cisplatin-induced p53 over-expression to 85 ± 3.3% of the control group (*p* < 0.05).

*In-vivo study*

*Effect of DNE on renal physiology and oxidative status*

No adverse effects were observed in the animals during the study. Cisplatin-induced renal function disruption was confirmed in the renal function index. BUN and CRE, and urine volume were significantly increased about 5-, 17-, and 2-fold, respectively, after cisplatin injection (Table 2). Meanwhile, DNE treatment improved the disrupted renal function index and urine volume, along with ameliorating body weight loss (*p* < 0.05).

The antioxidant activity of the DNE in cisplatin-induced renal injury was assessed by measuring GSH and MDA levels in the kidney (Table 2). Cisplatin treatment markedly increased the level of MDA in the kidney, whereas the tissue GSH level was significantly decreased when compared to the control (*p* < 0.05). In groups treated with DNE, the MDA level was decreased to about 50% (especially in the 500 mg/kg group) compared to the group treated with cisplatin alone. These groups also had increased GSH content (*p* < 0.05).

**Table 2:** Effect of *Dendrobium nobile* Lindl aqueous extract (DNE) on serum biochemical parameters, oxidative status and body weight in cisplatin-induced acute renal failure rats

Group	BUN (mg/dL)	CRE (mg/dL)	GSH (nmol/g tissue)	MDA (nmol/g tissue)	Body weight gains (g)	Urine Volume (mL)
Control	16.3±6.4	0.4±0.1	22.6±1.7	12.4±1.0	19±10	8±0
Cisplatin	83.0±12.7*	6.9±1.7*	7.8±1.3*	81.2±12.9*	-11±7*	16±0.6*
Captopril + cisplatin	18.0±4.2#	1.8±0.7#	16.5±4.0	14.6±8.0#	-10±6	10±1.2
DNE (100 mg/kg) + cisplatin	70.0±25.5*	3.4±1.1#	17.3±2.2	51.3±8.9	-7±1	14±1.7*
DNE (300 mg/kg) + cisplatin	15.0±2.0#	1.4±0.1#	23.6±3.9#	43.7±3.2	-3±2#	11±0.9
DNE (500 mg/kg) + cisplatin	12.0±7.07#	3.1±1.0#	24.6±2.4#	41.0±1.8#	-8±2*	8±1.1#

Rats (n=5/group) were given orally distilled water (DW), captopril (100 mg/kg) and DNE (100, 300 and 500 mg/kg) once daily for 28 days for each group. After 23 days of treatment, cisplatin (5 mg/kg) was intraperitoneally injected except in saline injected control. Values are expressed as mean ± SEM,

* *p* < 0.05, a significant difference in comparison with the control group,

# *p* < 0.05, a significant difference in comparison with the cisplatin alone treated group.

*Effect of DNE on histopathological alteration*

**Figure 4:**
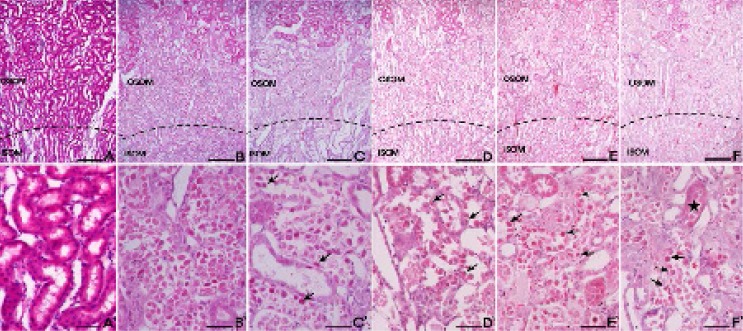
Effect of *Dendrobium nobile* Lindl extracts (DNE) on the cisplatin-treated rat kidney. H&E staining was used to assess tissue damage. (A) Control; (B) cisplatin alone; (C) captopril (100 mg/kg) pretreatment; (D) DNE (100 mg/kg) pretreatment; (E) DNE (300 mg/kg) pretreatment; (F) DNE (500 mg/kg) pretreatment group. All rats given orally each compound for 28 days. On day 23, cisplatin (5 mg/kg) was injected intraperitoneally to induce acute kidney injury. Tubular epithelial cells remained in the proximal tubules (arrows) in the S3 segments of proximal tubules of the outer stripe of the outer medulla (OSOM). The proximal tubules showed almost normal morphology (stars). Bar; 200 μm (A-F), 100 μm (A`-F`).

**Figure 5:**
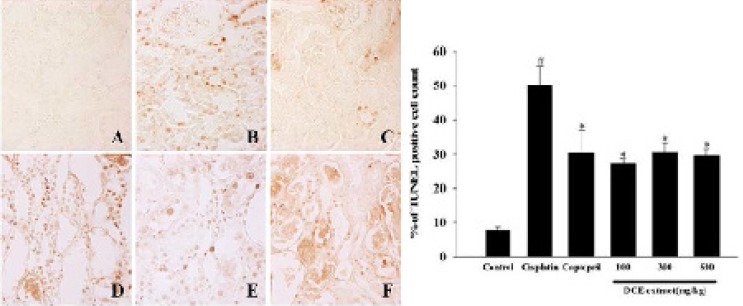
Effect of *Dendrobium nobile* Lindl extracts (DNE) on cisplatin-induced apoptosis in kidney. TUNEL staining was hired to assess apoptosis. (A) Control; (B) cisplatin alone; (C) captopril (100 mg/kg) pretreatment; (D) DNE (100 mg/kg) pretreatment; (E) DNE (300 mg/kg) pretreatment; (F) DNE (500 mg/kg) pretreatment group. All rats given orally each compound for 28 days. On day 23, cisplatin (5 mg/kg) was injected intraperitoneally to induce acute kidney injury. The presence of TUNEL-positive cells was measured by the image analyzer. Values are expressed as mean ± SEM. *p < 0.05, a significant difference in comparison with the group treated with cisplatin alone, #p < 0.05, a significant difference in comparison with the control group

The control group cells had an intact cytoplasm with a normal structure. In contrast, tubular detachments with a broad loss of the brush border were observed in the cells of the cisplatin alone group (Figure 4). In the DNE-pretreated group (especially at the concentration of 500 mg/kg), relatively well-preserved proximal tubules with reduced cell swelling were observed, although a slight desquamation and relative atrophy of the tubular epithelial cells were still present (Figure 4). Cisplatin-induced apoptosis in the kidney tissues was also detected using TUNEL staining (Figure 5). The percentage of TUNEL-positive cells markedly increased in the group treated with cisplatin alone. Although the decrement of apoptotic cell was not dose-dependent, the DNE-pretreated group showed a decreased proportion of apoptotic cells compared to the cisplatin-alone group (*p* < 0.05).

## Discussion

For a long time, fruits and medicinal plants that have an antioxidant effect have been used to reduce oxidative stress (21). Among these natural antioxidants, phenols and phenolic acids are considered to be the most active (22). Phenolic acids are a major class of polyphenols, which are widely distributed in the daily diet (23). Previous studies have demonstrated a relationship between the magnitude of antioxidant activity and the quantity of phenolic substances (24). Recently, a polysaccharide from the *Dendrobium* genus was reported to have an antioxidant effect with radical-scavenging activity (15, 25). Our study found that an aqueous extract of *D. nobile* contained phenolic acids including 4-hydroxybenzoic acid, vanillic acid, syringic acid, and ferulic acid. In DNE, 4-hydroxybenzoic acid was relatively abundant. It is well known that the structure of phenolic compounds is a key determinant of their radical scavenging activity and the antioxidant activity increase with increasing degree of hydroxylation (26). Although the antioxidant activity of phenolic acids depends on the number and position of the hydroxyl group, 4-hydroxybenzoic acid, a monohydroxybezoic acid, it is recognized as an effective hydroxyl radical scavenger due to its propensity for hydroxylation and the high reactivity of the hydroxyl radical (27). 

Many studies on the pathogenesis of cisplatin nephrotoxicity have focused on the oxidative stress induced by its direct toxicity and ROS known to induce apoptosis through the generation of hydroxyl radicals, and that an increase in the intracellular GSH concentration reduces the tissue damage caused by free radicals (10, 11, 28). GSH is a major antioxidant that protects cellular components from the ROS-derived damages (29). In this study, decreased GSH levels were observed in the cisplatin-treated cells, which might have resulted from the formation of cisplatin-GSH conjugates. In contrast, DNE-pretreated cells showed recovered GSH concentrations, almost comparable to the control cells, with reduced ROS production; this indicates a recovered antioxidant status. Several studies have reported that accumulated cisplatin in renal cells causes DNA damage and oxidative stress leading to apoptosis, which is related to the p53 signaling pathway (30, 31). p53 is a tumor-suppressor protein that acts in response to various forms of cellular damage derived from oxidative stress, and regulates the proliferative process (32). Moreover, cisplatin-induced p53 signal in caspase dependent apoptosis was confirmed by p53 null mouse model (33). In this study, DNE pretreatment down-regulated a cisplatin-induced p53 expression *in-vitro* and reduced apoptotic cells were found in the *in-vivo* TUNEL staining study.

In order to evaluate the protective effect of DNE against cisplatin-induced cell injury, the viability of the PK15 cells was tested using an MTT and crystal violet assay. Even though the recovery tendency was different, DNE pretreatment recovered cell viability. The different viability rates might be due to the different endpoints measured in each assay. The MTT assay is based on the function of mitochondrial dehydrogenase, while the crystal violet assay stains the nuclei of adherent cells (34). 

In the *in-vivo* model, DNE demonstrated a protective effect against cisplatin-induced AKI. Owing to its major side effects, cisplatin-induced nephrotoxicity model was frequently used to simulate AKI and renal failure (35). In this study, an elevation in serum BUN and CRE levels confirmed the presence of renal injury after cisplatin treatment. In contrast, the DNE-pretreated group showed decreased BUN and CRE levels with reduced weight loss after cisplatin injection. The S3 segment of the proximal tubule is an injury site specific to cisplatin-induced nephrotoxicity, and is where water uptake mainly occurs (36). Four weeks of DNE pretreatment decreased urine volume almost to control group levels.

The antioxidant effect of DNE in the rat model was consistent with the results of the *in-vitro* study. A decreased GSH and increased MDA levels were found in the group treated with cisplatin alone; the decreased level of antioxidants might lead to the elevation of lipid peroxidation, which was determined by MDA levels, which is an end product of lipid peroxidation. Meanwhile, the DNE treatment dose-dependently increased the GSH level. Lipid peroxidation could induce the cellular damages present in ROS-induced organ damage (37). In this study, MDA content in the kidney decreased in the DNE-pretreated group, which indicates decreased oxidative damage. Taken together, pretreatment with DNE attenuated the cisplatin-induced progressive oxidative damage of the kidney by enhancing the antioxidant marker (GSH) and lowering the oxidative stress marker (MDA).

A histopathological study was performed to confirm the protective effects of DNE in the kidney tissue. Twenty-eight days of DNE treatment (especially at a concentration of 500 mg/kg) reduced cisplatin-induced histopathological alterations in the region of the proximal tubule. Moreover, DNE pretreatment markedly the decreased TUNEL-positive cell number although the effect was not dose-dependent. 

## Conclusions

In the present study, DNE, which contains phenolic acids, was found to have a protective effect against cisplatin-induced nephrotoxicity both *in-vitro* and *in-vivo*. DNE treatment enhanced cell viability and ameliorated renal damage by decreasing oxidative stress. Thus, DNE could potentially be used in the prevention of AKI. Further studies are needed to determine the therapeutic window of DNE for clinical application.

## References

[1] Dennen P, Parikh CR (2007). Biomarkers of acute kidney injury: can we replace serum creatinine? Clin. Nephrol.

[2] Yao X, Panichpisal K, Kurtzman N, Nugent K (2007). Cisplatin nephrotoxicity: a review. Am. J. Med. Sci.

[3] Arany I, Safirstein RL (2003). Cisplatin nephrotoxicity. Semin. Nephrol.

[4] Ali BH, Al Moundhri MS (2006). Agents ameliorating or augmenting the nephrotoxicity of cisplatin and other platinum compounds: a review of some recent research. Food Chem. Toxicol.

[5] Baliga R, Ueda N, Walker PD, Shah SV (1999). Oxidant mechanisms in toxic acute renal failure. Drug Metab. Rev.

[6] Townsend DM, Hanigan MH (2002). Inhibition of gamma-glutamyltranspeptidase or cysteine S-conjugate beta-lyase activity blocks the nephrotoxicity of cisplatin in mice. J. Pharmacol. Exp. Ther.

[7] Matsushima H, Yonemura K, Ohishi K, Hishida A (1998). The role of oxygen free radicals in cisplatin-induced acute renal failure in rats. J. Lab. Clin. Med.

[8] Santos NA, Bezerra CS, Martins NM, Curti C, Bianchi ML, Santos AC (2008). Hydroxyl radical scavenger ameliorates cisplatin-induced nephrotoxicity by preventing oxidative stress, redox state unbalance, impairment of energetic metabolism and apoptosis in rat kidney mitochondria. Cancer Chemother. Pharmacol.

[9] Atessahin A, Yilmaz S, Karahan I, Ceribasi AO, Karaoglu A (2005). Effects of lycopene against cisplatin-induced nephrotoxicity and oxidative stress in rats. Toxicology.

[10] Behling EB, Sendao MC, Francescato HD, Antunes LM, Costa RS, Bianchi ML (2006). Comparative study of multiple dosage of quercetin against cisplatininduced nephrotoxicity and oxidative stress in rat kidneys. Pharmacol. Rep.

[11] Kim TW, Song IB, Lee HK, Lim JH, Cho ES, Son HY, Yun HI (2012). Platycodin D, a triterpenoid sapoinin from Platycodon grandiflorum, ameliorates cisplatin-induced nephrotoxicity in mice. Food Chem. Toxicol.

[12] Chen X, Guo S (2000). Advances in the research of constituents and pharmacology of Dendrobium. Nat. Prod. Res. Dev.

[13] Yang H, Sung SH, Kim YC (2007). Antifibrotic phenanthrenes of Dendrobium nobile stems. J. Nat. Prod.

[14] Zhang X, Xu JK, Wang J, Wang NL, Kurihara H, Kitanaka S, Yao XS (2007). Bioactive bibenzyl derivatives and fluorenones from Dendrobium nobile. J. Nat. Prod.

[15] Luo A, He X, Zhou S, Fan Y, Luo A, Chun Z (2010). Purification, composition analysis and antioxidant activity of the polysaccharides from Dendrobium nobile Lindl. Carbohydr. Polym.

[16] Wang JH, Luo JP, Zha XQ, Feng BJ (2010). Comparison of antitumor activities of different polysaccharide fractions from the stems of Dendrobium nobile Lindl. Carbohydr. Polym.

[17] Clément MV, Hirpara JL, Chawdhury SH, Pervaiz S (1998). Chemopreventive agent resveratrol, a natural product derived from grapes, triggers CD95 signaling-dependent apoptosis in human tumor cells. Blood.

[18] LeBel CP, Bondy SC (1990). Sensitive and rapid quantitation of oxygen reactive species formation in rat synaptosomes. Neurochem. Int.

[19] dos Santos NAG, Rodrigues MAC, Martins NM, dos Santos AC (2012). Cisplatin-induced nephrotoxicity and targets of nephroprotection: an update. Arch. Toxicol.

[20] El-Sayed ESM, Abd-Ellah MF, Attia SM (2008). Protective effect of captopril against cisplatin-induced nephrotoxicity in rats. Pak. J. Pharm. Sci.

[21] Rice-Evans CA, Miller NJ, Bolwell PG, Bramley PM, Pridham JB (1995). The relative antioxidant activities of plant-derived polyphenolic flavonoids. Free Radic. Res.

[22] Naczk M, Shahidi F (2006). Phenolics in cereals, fruits and vegetables: Occurrence, extraction and analysis. J. Pharmaceut. Biomed.

[23] Piazzon A, Vrhovsek U, Masuero D, Mattivi F, Mandoj F, Nardini M (2012). Antioxidant activity of phenolic acids and their metabolites: synthesis and antioxidant properties of the sulfate derivatives of ferulic and caffeic acids and of the acyl glucuronide of ferulic acid. J. Agric. Food Chem.

[24] Büyükbalci A, El SN (2008). Determination of in-vitro antidiabetic effects, antioxidant activities and phenol contents of some herbal teas. Plant Food Hum. Nutr.

[25] Bao SH, Zha XQ, Hao J, Luo JP (2009). In-vitro antioxidant activity of polysaccharides with different molecular weights from Dendrobium candidum. Food Sci.

[26] Balasundram N, Sundram K, Samman S (2006). Phenolic compounds in plants and agri-industrial by-products: Antioxidant activity, occurrence, and potential uses. Food Chem.

[27] Rice-Evans, Catherine A, Nicholas JM, George P (1996). Structure-antioxidant activity relationships of flavonoids and phenolic acids. Free Rad. Bio. Med.

[28] Simon HU, Haj-Yehia A, Levi-Schaffer F (2000). Role of reactive oxygen species (ROS) in apoptosis induction. Apoptosis.

[29] Kaplowitz N (1981). The importance and regulation of hepatic glutathione. Yale J. Biol. Med.

[30] Bragado P, Armesilla A, Silva A, Porras A (2007). Apoptosis by cisplatin requires p53 mediated p38α MAPK activation through ROS generation. Apoptosis.

[31] Jiang M, Wei Q, Pabla N, Dong G, Wang CY, Yang T, Dong Z (2007). Effects of hydroxyl radical scavenging on cisplatin-induced p53 activation, tubular cell apoptosis and nephrotoxicity. Biochem. Pharm.

[32] Vousden KH, Lane DP (2007). p53 in health and disease. Nat. Rev. Mol. Cell Biol.

[33] Yang C, Kaushal V, Haun RS, Seth R, Shah SV, Kaushal GP (2008). Transcriptional activation of caspase-6 and-7 genes by cisplatin-induced p53 and its functional significance in cisplatin nephrotoxicity. Cell Death Differ.

[34] Ramezanpour M, da Silva BK, Sanderson BJS (2012). Differential susceptibilities of human lung, breast and skin cancer cell lines to killing by five sea anemone venoms. J. Venom Anim. Toxins.

[35] Singh AP, Junemann A, Muthuraman A, Jaggi AS, Singh N, Grover K, Dhawan R (2012). Animal models of acute renal failure. Pharmacol. Rep.

[36] Dobyan DC, Levi J, Jacobs C, Kosek J, Weiner MW (1980). Mechanism of cisplatinum nephrotoxicity: II Morphologic observations. J. Pharmacol. Exp. Ther.

[37] Blokhina O, Virolainen E, Fagerstedt KV (2003). Antioxidants, oxidative damage and oxygen deprivation stress: a review. Ann. Bot.

